# Neurolymphomatosis in patient with Adult T-cell leukaemia/lymphoma (ATLL)

**DOI:** 10.1186/1742-4690-12-S1-P29

**Published:** 2015-08-28

**Authors:** Maki Yoshimitsu, Arata Hitoshi, Matsuura Eiji, Izumo Shuji, Takashima Hiroshi

**Affiliations:** 1 Department of Neurology and Geriatrics, Kagoshima University Graduate School of Medical and Dental Sciences, Japan; 2 Department of Molecular Pathology, Center for Chronic Viral Diseases, Kagoshima University Graduate School of Medical and Dental Sciences, Japan

## 

Neurolymphomatosis is a rare manifestation of lymphoma characterized by infiltration of the peripheral nerves, leading to neuropathy. Although recent imaging studies such as PET study make it possible to diagnose neurolymphomatosis in the patient with lymphoma, the histochemical studies on a case with neurolymphomatosis by ATLL are still rare. We here report a patient presenting neurolymphomatosis of ATLL diagnosed by a histochemical study of biopsied sural nerve. A 74-year-old man presented a skin rush of left hand and was diagnosed with smoldering ATLL. A physical examination revealed slight weakness of the muscle of the extremities and sensory deficits with painful paresthesia. F-18 fluorodeoxy-glucose positron emission tomography (FDG-PET) demonstrated a cervical lesion at cervical lymph node but not a nerve root lesion suggesting neurolymphomatosis. The histochemical study of biopsied skin showed the CD4, CD8, and CD25 triple-positive cells in epidermal Pautrier microabscesses. Normal structure of lymph node was not seen in lymph node and was replaced with fatty tissue. Medium-sized atypical lymphocytic cells were scatterly noted with small lymphocytes. Those atypical lymphocytic cells had hyperchromatic pleomorphic nuclei and prominent nucleoli. While nerve conduction study revealed severe sensory dominant peripheral neuropathy, the density of myelinatied fibers was relatively preserved in biopsied sural nerve. The Histological examination showed a number of atypical lymphocytes broadly infiltrating perineurial connective tissue and subperineurium. The patient was treated with intravenous injection of methylpredonisolone. Although the treatment effects on the pain of the extremities and motor ability of the upper extremities just for short period after the treatment, sensory and motor symptoms progressed worse. A histological study of peripheral nerve is still useful for a diagnosis of neurolymphomatosis.

